# The effectiveness and safety of paediatric prehospital pain management: a systematic review

**DOI:** 10.1186/s13049-021-00974-3

**Published:** 2021-12-11

**Authors:** Yonas Abebe, Fredrik Hetmann, Kacper Sumera, Matt Holland, Trine Staff

**Affiliations:** 1 Department of Emergency and Critical Care Nursing, St. Paul’s Hospital Millennium Medical College, Addis Ababa, Ethiopia; 2 Bachelor Programme in Paramedics, Institute of Nursing and Health Promotion, Faculty of Health Science, Oslo Metropolitan University, Oslo, Norway; 3 Edge Hill University, Ormskirk, UK; 4 Library and Knowledge Services for NHS Ambulance Services in England, Bolton, UK

**Keywords:** Children, Paediatrics, Prehospital, Ambulance, Analgesia, Pain management, Fentanyl, Morphine, Methoxyflurane, Ketamine

## Abstract

**Background:**

Clinically meaningful pain reduction with respect to severity and the adverse events of drugs used in prehospital pain management for children are areas that have not received sufficient attention. The present systematic review therefore aims to perform a comprehensive search of databases to examine the preferable drugs for prehospital pain relief in paediatric patients with acute pain, irrespective of aetiology.

**Methods:**

The systematic review includes studies from 2000 and up to 2020 that focus on children’s prehospital pain management. The study protocol is registered in PROSPERO with registration no. CRD42019126699. Pharmacological pain management using any type of analgesic drug and in all routes of administration was included. The main outcomes were (1) measurable pain reduction (effectiveness) and (2) no occurrence of any serious adverse events. Searches were conducted in PubMed, Medline, Embase, CINAHL, Epistemonikos and Cochrane library. Finally, the risk of bias was assessed using the Joanna Briggs Institute (JBI) checklist and a textual narrative analysis was performed due to the heterogeneity of the results.

**Results:**

The present systematic review on the effectiveness and safety of analgesic drugs in prehospital pain relief in children identified a total of eight articles. Most of the articles reviewed identified analgesic drugs such as fentanyl (intranasal/IV), morphine (IV), methoxyflurane (inhalational) and ketamine (IV/IM). The effects of fentanyl, morphine and methoxyflurane were examined and all of the included analgesic drugs were evaluated as effective. Adverse events of fentanyl, methoxyflurane and ketamine were also reported, although none of these were considered serious.

**Conclusion:**

The systematic review revealed that fentanyl, morphine, methoxyflurane and combination drugs are effective analgesic drugs for children in prehospital settings. No serious adverse events were reported following the administration of fentanyl, methoxyflurane and ketamine. Intranasal fentanyl and inhalational methoxyflurane seem to be the preferred drugs for children in pre-hospital settings due to their ease of administration, similar effect and safety profile when compared to other analgesic drugs. However, the level of evidence (LOE) in the included studies was only three or four, and further studies are therefore necessary.

## Background

Prehospital care providers have traditionally focused on time-sensitive acute illness and major traumas, which represent only a small number of patients. However, in the prehospital setting, large groups of patients experience a variety of illnesses and injuries with frequent symptoms and signs, including pain [1]. It is thought that effective analgesia is one of the top outcome measures of prehospital care [2]. It is also considered among the top factors in the satisfaction of the patients’ family [3]. The prevalence of acute pain in prehospital settings ranges from 42 to 53% [4–6].

Previous studies of children in prehospital care show that although the pain was documented in the ambulance records, the level of pain was not assessed in 66–96% of cases [5, 7]. Previous findings also show that 52–88% of children in prehospital setups did not receive pain medication despite having moderate to severe pain [5, 8]. The most common reasons for not providing adequate pain medication to children in prehospital settings include fearing side effects, difficulties with intravenous (IV) line access, being under five years of age, the lack of a pain assessment and the assumption that children need less analgesia than adults [9–12].

The evidence, however, indicates that adequate prehospital pain management in children relieves suffering, contributes to timely emergency department (ED) analgesia, prevents chronic pain and improves recovery [13, 14]. Conversely, inadequate pain management harms children’s development and increases morbidity and mortality [15, 16]. The negative effect of inadequate pain management could also extend to fear of medical care or medical/medication over-use in adulthood [17].

In prehospital pain medication for children, clinically meaningful reductions of pain severity and evaluations of adverse events of drugs for different age groups are areas that lack sufficient investigation. A few small-scale and systematic reviews of prehospital pain management have been conducted, but these either concerned the adult age group, trauma aetiologies or were specific to one analgesic agent only [18–20]. Because of the lack of high-level evidence, there are no clear guidelines regarding the choice of drug, recommended dose or which route of administration is preferable for prehospital pain management of children, irrespective of aetiology [12, 21]. The current original systematic review aims to examine the effectiveness and safety of analgesic drugs for prehospital pain relief in paediatric patients with acute pain of any cause.

## Methods

This systematic review was conducted in accordance with the Preferred Reporting Items for Systematic Reviews and Meta-analyses (PRISMA) guidelines [22]. The review question, outcomes, inclusion criteria and methods of analysis were predefined, and the protocol was registered in the International Prospective Register of Systematic Reviews (PROSPERO), with registration no. CRD42019126699 [23].

### Review question and types of participants

The main research question for this systematic review was, ‘What are the preferred drugs for prehospital pain relief in paediatric patients with acute pain?’. The study participants were children under the age of eighteen with acute pain (i.e. a sudden pain lasting less than 3 months) in the prehospital setting. A prehospital setting is defined here as a place where any acute medical care is provided by ambulance care providers before the patient arrives at the hospital. In this review, preferable analgesic drugs were determined by the effectiveness and safety of the drugs. The primary outcome of this review concerned the effectiveness of the analgesic drugs employed. The drugs’ effectiveness was defined as a clinically meaningful pain reduction as measured by a reduction of two or more points from the initial pain severity score after the administration of analgesic drugs based on standardised clinical pain assessment tools [24]. The secondary outcome concerned safety as defined by no occurrence of any serious adverse events in the prehospital setting after analgesic administration. The U.S. Food and Drug Administration (FDA) defines a serious adverse event as any serious undesirable experiences such as death, substantial risk of dying (life-threatening), hospitalisation, disability or permanent damage, congenital anomaly, required intervention to prevent permanent impairment and other serious medical events associated with the use of a medical product in a patient [25].

### Eligibility criteria

The current review included all studies that focus on children’s prehospital pain management in low-, middle- and high-income countries. We included any mixed age group studies if there was a separate analysis for children under eighteen years of age. Since knowing the standalone effect of other non-pharmacological pain managements is difficult, only studies that addressed pharmacological pain management with any type of analgesic drugs in all routes of administration were included. Pain evaluation papers were included if pharmacological pain interventions were integrated. To examine all types of analgesic drugs that are used, we included all qualifications of prehospital care providers despite variation in their training, scope and expertise in the different prehospital setups. The publications included in this review comprised randomised control trials, non-randomised control studies, a cohort with control groups, interrupted time series, cross-sectional and case series studies.

Studies that examined chronic pain were excluded because the patient’s response to chronic pain management is different when compared to acute pain management [26]. As with the medical setup, the patients’ characteristics and the level of the responders’ training varied, studies that report on any sort of out-of-hospital pain treatments given by non-ambulance service providers, ambulance care while in medical transfers and in-hospital transfers were excluded. Pain management given in fixed healthcare facilities and/or by any other non-healthcare professionals was also excluded. As the primary and secondary outcomes of this review concerned the effectiveness and safety of analgesic drugs, studies that did not produce findings on either of these outcomes were excluded. In addition, qualitative studies, case reports, guidelines, continuing professional development (CPDs), letters to editors, service evaluation, conference abstracts and abstracts that did not have full text were also excluded.

### Search strategy

The search strategy was developed by the four authors (YA, TS, FH and KS) who are the subject specialists and was peer-reviewed by another author (MH) who is a research librarian. Studies were identified through electronic database searches including PubMed, Ovid Medline (1946 to 15 December 2020), Ovid Embase (1974 to 15 December 2000), CINAHL (Ebsco), Epistemonikos and Cochrane library. The relevant medical subject headings (MeSH terms) and keywords used for the systematic search strategy are presented in Table 1. The searches were limited as regards publication year (the past 20 years from 2000 to 2020) and language (English, Danish, Norwegian or Swedish). The search strategy for each database is presented in Tables 2, 3, 4, 5, 6,  7. All independent reviewers performed the literature searches from all included databases and imported these to the Covidence software. The last searches were rerun by the research librarian (MH) on 16 December 2020. In addition, a hand search was conducted of the reference lists of the studies included in the current systematic review and systematic review reports concerning a similar topic, which resulted in the identification of one additional article.

**Table 1:** The MeSH terms and keywords according to the modified PICO

Modified PICO	Medline (Ovid)	Embase (Ovid)	PubMed	CINAHL (Ebsco)	Cochrane	Epistemonikos	Keywords
Patient (children)	Child or infant or adolescent or pediatrics	Child or juvenile or infant or preschool child or school child or toddler or adolescent or newborn or pediatrics or pediatric emergency medicine	Child or infant or adolescent or pediatrics	Child or infant or adolescence or pediatrics	Child or adolescent or infant or pediatrics	Only key word	Infant* or newborn* or child* or "preschool child*" or juvenile* or preschool* or adolescent* or pediatric* or paediatric* or "young people" or "young person"
	**AND**	**AND**	**AND**	**AND**	**AND**	**AND**	**AND**
Population (prehospital)	Ambulances or emergency medical services or advanced trauma life support care or transportation of patients or emergency medicine or emergency responders or emergency medical technicians or emergency nursing	Ambulance or emergency health service or rescue personnel or emergency nursing or emergency medicine or pediatric emergency medicine	Emergency medical services or emergency medicine or emergency medical technicians	Prehospital care or emergency medical services or transportation of patients or ambulances or emergency medicine or emergency medical technicians	Emergency medical services or ambulances or emergency medicine or emergency medical technicians or emergency nursing	Only key word	Ambulance* or "transportation of patient*" or "emergency service*" or "emergency medical service*" or ems or "emergency health service*" or "prehospital care" or prehospital* or pre-hospital* or "out of hospital" or "out of hospital care" or "emergency medicine" or "pediatric emergency medicine" or "paediatric emergency medicine" or "emergency responder*" or "first responder*" or "emergency medical technician*" or "emergency technician*" or "emergency practitioner*" or " emergency medical practitioner*" or "emergency care practitioner*" or emt or "rescue personnel*" or "emergency nurse*" or paramedic*
	**AND**	**AND**	**AND**	**AND**	**AND**	**AND**	**AND**
Intervention (pharmacologic pain management) or Outcome (pain reduction/ adverse event)	Analgesia or analgesics or nerve block or fentanyl or ketamine or morphine or acetaminophen or drug administration routes or pain measurement	Analgesia or analgesic agent or nerve block or fentanyl or ketamine or morphine or paracetamol or drug administration route or pain measurement or numeric rating scale or pain assessment	Pain management or analgesia or analgesics or nerve block or fentanyl or ketamine or morphine or acetaminophen or drug administration routes or pain measurement	Pain management or analgesia or analgesics or nerve block or acetaminophen or drug administration routes or pain measurement	Pain management or analgesia or analgesics or nerve block or fentanyl or ketamine or morphine or acetaminophen or drug administration routes or pain measurement	Only key word	"Pain management*" or "pain treatment*" or "pain intervention*" or analgesi* or analgaesi* or "analgetic agent*" or "analgesic drug*" or anodynes or antinociceptive or nsaids or paracetamol or ketamine or fentanyl or "nitrose oxide" or methoyloxine or morphine or opoid or non-opoid or narcotics or non-narcotics or "regional nerve block*" or "per oral" or peroral or intravanous or "intra venous" or iv or intramuscular or "intra muscular" or "intra nasal" or intranasal or inhalational or "pain reduction" or "pain relief" or oligoanalgesia or "pain intensity" or "pain assessment" or "pain measurement" or effectiveness or "effect management" or "side effect*" or "adverse effect*" or "adverse event*"
Limit	Publication year (the past 20 years from 2000 to 2020), language (English, Danish, Norwegian, and Swedish)

**Table 2:** Medline Search Strategy

1. (infant* or newborn* or child* or "preschool child*" or juvenile* or preschool* or adolescent* or pediatric* or paediatric* or "young people" or "young person").tw,kw,kf 2. exp child/ or exp infant/ 3. Adolescent/ 4. exp Pediatrics/ 5. 1 or 2 or 3 or 4 6. (ambulance* or "transportation of patient*" or "emergency service*" or "emergency medical service*" or EMS or "emergency health service*" or "prehospital care" or prehospital* or pre-hospital* or "out of hospital" or "out of hospital care" or "emergency medicine" or "pediatric emergency medicine" or "paediatric emergency medicine" or "emergency responder*" or "first responder*" or "emergency medical technician*" or "emergency technician*" or "emergency practitioner*" or " emergency medical practitioner*" or "emergency care practitioner*" or EMT or "rescue personnel*" or "emergency nurse*" or paramedic*).tw,kw,kf 7. Ambulances/ 8. emergency medical services/ or advanced trauma life support care/ or "transportation of patients"/ 9. exp Emergency Medicine/ 10. emergency responders/ or emergency medical technicians/ 11. Emergency Nursing/ 12. 6 or 7 or 8 or 9 or 10 or 11 13. ("pain management*" or "pain treatment*" or "pain intervention*" or analgesi* or analgaesi* or "analgetic agent*" or "analgesic drug*" or anodynes or antinociceptive or NSAIDs or paracetamol or ketamine or fentanyl or "nitrose oxide" or methoyloxine or morphine or opoid or non-opoid or narcotics or non-narcotics or "regional nerve block*" or "per oral" or peroral or intravanous or "intra venous" or IV or intramuscular or "intra muscular" or "intra nasal" or intranasal or inhalational or "pain reduction" or "pain relief" or oligoanalgesia or "pain intensity" or "pain assessment" or "pain measurement" or effectiveness or "effect management" or "side effect*" or "adverse effect*" or "adverse event*").tw,kw,kf 14. Pain Management/ 15. Analgesia/ 16. exp Analgesics/ 17. Nerve Block/ 18. exp Fentanyl/ 19. Ketamine/ 20. Morphine/ 21. Acetaminophen/ 22. exp Drug Administration Routes/ 23. Pain Measurement/ 24. 13 or 14 or 15 or 16 or 17 or 18 or 19 or 20 or 21 or 22 or 23 25. 5 and 12 and 24 26. limit 25 to (humans and yr = "2000—2020" and "all child (0 to 18 years)" and (danish or english or norwegian or swedish))

**Table 3:** Embase Search Strategy

1. (infant* or newborn* or child* or "preschool child*" or juvenile* or preschool* or adolescent* or pediatric* or paediatric* or "young people" or "young person").tw,kw 2. child/ or juvenile/ or infant/ or preschool child/ or school child/ or toddler/ 3. adolescent/ 4. newborn/ 5. pediatrics/ or pediatric emergency medicine/ 6. 1 or 2 or 3 or 4 or 5 7. (ambulance* or "transportation of patient*" or "emergency service*" or "emergency medical service*" or EMS or "emergency health service*" or "prehospital care" or prehospital* or pre-hospital* or "out of hospital" or "out of hospital care" or "emergency medicine" or "pediatric emergency medicine" or "paediatric emergency medicine" or "emergency responder*" or "first responder*" or "emergency medical technician*" or "emergency technician*" or "emergency practitioner*" or " emergency medical practitioner*" or "emergency care practitioner*" or EMT or "rescue personnel*" or "emergency nurse*" or paramedic*).tw,kw 8. ambulance/ 9. emergency health service/ 10. exp rescue personnel/ 11. emergency nursing/ 12. emergency medicine/ or pediatric emergency medicine/ 13. 7 or 8 or 9 or 10 or 11 or 12 14. ("pain management*" or "pain treatment*" or "pain intervention*" or analgesi* or analgaesi* or "analgetic agent*" or "analgesic drug*" or anodynes or antinociceptive or NSAIDs or paracetamol or ketamine or fentanyl or "nitrose oxide" or methoyloxine or morphine or opoid or non-opoid or narcotics or non-narcotics or "regional nerve block*" or "per oral" or peroral or intravanous or "intra venous" or IV or intramuscular or "intra muscular" or "intra nasal" or intranasal or inhalational or "pain reduction" or "pain relief" or oligoanalgesia or "pain intensity" or "pain assessment" or "pain measurement" or effectiveness or "effect management" or "side effect*" or "adverse effect*" or "adverse event*").tw,kw 15. analgesia/ 16. analgesic agent/ 17. nerve block/ 18. fentanyl/ 19. ketamine/ 20. morphine/ 21. paracetamol/ 22. exp drug administration route/ 23. pain measurement/ or numeric rating scale/ 24. pain assessment/ 25. 14 or 15 or 16 or 17 or 18 or 19 or 20 or 21 or 22 or 23 or 24 26. 6 and 13 and 25 27. limit 26 to (human and (danish or english or norwegian or swedish) and yr = "2000—2020" and (infant or child or preschool child < 1 to 6 years > or school child < 7 to 12 years > or adolescent < 13 to 17 years >))

**Table 4:** PubMed Search Strategy

#1. infant*[Title/Abstract] OR newborn*[Title/Abstract] OR child*[Title/Abstract] OR "preschool child*"[Title/Abstract] OR juvenile*[Title/Abstract] OR preschool*[Title/Abstract] OR adolescent*[Title/Abstract] OR pediatric*[Title/Abstract] OR paediatric*[Title/Abstract] OR "young people"[Title/Abstract] OR "young person"[Title/Abstract] #2. "Child"[Mesh] OR "Infant"[Mesh] OR "Adolescent"[Mesh] OR "Pediatrics"[Mesh] #3. #1 OR #2 #4. ambulance*[Title/Abstract] OR "transportation of patient*"[Title/Abstract] OR "emergency service*"[Title/Abstract] OR "emergency medical service*"[Title/Abstract] OR EMS[Title/Abstract] OR "emergency health service*"[Title/Abstract] OR "prehospital care"[Title/Abstract] OR prehospital*[Title/Abstract] OR pre-hospital*[Title/Abstract] OR "out of hospital"[Title/Abstract] OR "out of hospital care"[Title/Abstract] OR "emergency medicine"[Title/Abstract] OR "pediatric emergency medicine"[Title/Abstract] OR "paediatric emergency medicine"[Title/Abstract] OR "emergency responder*"[Title/Abstract] OR "first responder*"[Title/Abstract] OR "emergency medical technician*"[Title/Abstract] OR "emergency technician*"[Title/Abstract] OR "emergency practitioner*"[Title/Abstract] OR " emergency medical practitioner*"[Title/Abstract] OR "emergency care practitioner*"[Title/Abstract] OR EMT[Title/Abstract] OR "rescue personnel*"[Title/Abstract] OR "emergency nurse*"[Title/Abstract] OR paramedic*[Title/Abstract] #5. "Emergency Medical Services"[Mesh] OR "Emergency Medicine"[Mesh] OR "Emergency Medical Technicians"[Mesh] #6. #4 OR #5 #7. "pain management*"[Title/Abstract] OR "pain treatment*"[Title/Abstract] OR "pain intervention*"[Title/Abstract] OR analgesi*[Title/Abstract] OR analgaesi*[Title/Abstract] OR "analgetic agent*"[Title/Abstract] OR "analgesic drug*"[Title/Abstract] OR anodynes[Title/Abstract] OR antinociceptive[Title/Abstract] OR NSAIDs[Title/Abstract] OR paracetamol[Title/Abstract] OR ketamine[Title/Abstract] OR fentanyl[Title/Abstract] OR "nitrose oxide"[Title/Abstract] OR methoyloxine[Title/Abstract] OR morphine[Title/Abstract] OR opoid[Title/Abstract] OR non-opoid[Title/Abstract] OR narcotics[Title/Abstract] OR non-narcotics[Title/Abstract] OR "regional nerve block*"[Title/Abstract] OR "per oral"[Title/Abstract] OR peroral[Title/Abstract] OR intravanous[Title/Abstract] OR "intra venous"[Title/Abstract] OR IV[Title/Abstract] OR intramuscular[Title/Abstract] OR "intra muscular"[Title/Abstract] OR "intra nasal"[Title/Abstract] OR intranasal[Title/Abstract] OR inhalational[Title/Abstract] OR "pain reduction"[Title/Abstract] OR "pain relief"[Title/Abstract] OR oligoanalgesia[Title/Abstract] OR "pain intensity"[Title/Abstract] OR "pain assessment"[Title/Abstract] OR "pain measurement"[Title/Abstract] OR effectiveness[Title/Abstract] OR "effect management"[Title/Abstract] OR "side effect*"[Title/Abstract] OR "adverse effect*"[Title/Abstract] OR "adverse event*"[Title/Abstract] #8. "Pain Management"[Mesh] OR "Analgesia"[Mesh] OR "Analgesics"[Mesh] OR "Nerve Block"[Mesh] OR "Fentanyl"[Mesh] OR "Ketamine"[Mesh] OR "Morphine"[Mesh] OR "Acetaminophen"[Mesh] OR "Drug Administration Routes"[Mesh] OR "Pain Measurement"[Mesh] #9. #7 OR #8 #10. #3 AND #6 AND #9 #11. #3 AND #6 AND #9 Filters: Humans, Danish, English, Norwegian, Swedish, Child: birth-18 years, from 2000—2020

**Table 5:** CINAHL Search Strategy

S1. TI (infant* or newborn* or child* or "preschool child*" or juvenile* or preschool* or adolescent* or pediatric* or paediatric* or "young people" or "young person") OR AB ( infant* or newborn* or child* or "preschool child*" or juvenile* or preschool* or adolescent* or pediatric* or paediatric* or "young people" or "young person") S2. (MH "Child + ") OR (MH "Infant + ") S3. (MH "Adolescence") S4. (MH "Pediatrics") S5. S1 OR S2 OR S3 OR S4 S6. TI ( ambulance* or "transportation of patient*" or "emergency service*" or "emergency medical service*" or EMS or "emergency health service*" or "prehospital care" or prehospital* or pre-hospital* or "out of hospital" or "out of hospital care" or "emergency medicine" or "pediatric emergency medicine" or "paediatric emergency medicine" or "emergency responder*" or "first responder*" or "emergency medical technician*" or "emergency technician*" or "emergency practitioner*" or " emergency medical practitioner*" or "emergency care practitioner*" or EMT or "rescue personnel*" or "emergency nurse*" or paramedic*) OR AB ( ambulance* or "transportation of patient*" or "emergency service*" or "emergency medical service*" or EMS or "emergency health service*" or "prehospital care" or prehospital* or pre-hospital* or "out of hospital" or "out of hospital care" or "emergency medicine" or "pediatric emergency medicine" or "paediatric emergency medicine" or "emergency responder*" or "first responder*" or "emergency medical technician*" or "emergency technician*" or "emergency practitioner*" or " emergency medical practitioner*" or "emergency care practitioner*" or EMT or "rescue personnel*" or "emergency nurse*" or paramedic*) S7. (MH "Prehospital Care") S8. (MH "Emergency Medical Services") OR (MH "Transportation of Patients") S9. (MH "Ambulances") S10. (MH "Emergency Medicine") S11. (MH "Emergency Medical Technicians") S12. S6 OR S7 OR S8 OR S9 OR S10 OR S11 S13. TI ( "pain management*" or "pain treatment*" or "pain intervention*" or analgesi* or analgaesi* or "analgetic agent*" or "analgesic drug*" or anodynes or antinociceptive or NSAIDs or paracetamol or ketamine or fentanyl or "nitrose oxide" or methoyloxine or morphine or opoid or non-opoid or narcotics or non-narcotics or "regional nerve block*" or "per oral" or peroral or intravanous or "intra venous" or IV or intramuscular or "intra muscular" or "intra nasal" or intranasal or inhalational or "pain reduction" or "pain relief" or oligoanalgesia or "pain intensity" or "pain assessment" or "pain measurement" or effectiveness or "effect management" or "side effect*" or "adverse effect*" or "adverse event*") OR AB ( "pain management*" or "pain treatment*" or "pain intervention*" or analgesi* or analgaesi* or "analgetic agent*" or "analgesic drug*" or anodynes or antinociceptive or NSAIDs or paracetamol or ketamine or fentanyl or "nitrose oxide" or methoyloxine or morphine or opoid or non-opoid or narcotics or non-narcotics or "regional nerve block*" or "per oral" or peroral or intravanous or "intra venous" or IV or intramuscular or "intra muscular" or "intra nasal" or intranasal or inhalational or "pain reduction" or "pain relief" or oligoanalgesia or "pain intensity" or "pain assessment" or "pain measurement" or effectiveness or "effect management" or "side effect*" or "adverse effect*" or "adverse event*") S14. (MH "Pain Management") S15. (MH "Analgesia") S16. (MH "Analgesics + ") S17. (MH "Nerve Block") S18. (MH "Acetaminophen") S19. (MH "Drug Administration Routes + ") S20. (MH "Pain Measurement") S21. S13 OR S14 OR S15 OR S16 OR S17 OR S18 OR S19 OR S20 S22. S5 AND S12 AND S21 S23. S5 AND S12 AND S21 Limiters—Age Groups: Infant, Newborn: birth-1 month, Infant: 1–23 months, Child, Preschool: 2–5 years, Child: 6–12 years, Adolescent: 13–18 years, All Infant, All Child; Published Date: 20,000,101–20,201,231; Human; Language: Danish, English, Norwegian, Swedish

**Table 6:** Cochrane Library Search Strategy

#1. (infant* or newborn* or child* or "preschool child*" or juvenile* or preschool* or adolescent* or pediatric* or paediatric* or "young people" or "young person"):ti,ab,kw #2. MeSH descriptor: [Child] explode all trees #3. MeSH descriptor: [Adolescent] explode all trees #4. MeSH descriptor: [Infant] explode all trees #5. MeSH descriptor: [Pediatrics] explode all trees #6. #1 or #2 or #3 or #4 or #5 #7. (ambulance* or "transportation of patient*" or "emergency service*" or "emergency medical service*" or EMS or "emergency health service*" or "prehospital care" or prehospital* or pre-hospital* or "out of hospital" or "out of hospital care" or "emergency medicine" or "pediatric emergency medicine" or "paediatric emergency medicine" or "emergency responder*" or "first responder*" or "emergency medical technician*" or "emergency technician*" or "emergency practitioner*" or " emergency medical practitioner*" or "emergency care practitioner*" or EMT or "rescue personnel*" or "emergency nurse*" or paramedic*):ti,ab,kw #8. MeSH descriptor: [Emergency Medical Services] explode all trees #9. MeSH descriptor: [Ambulances] in all MeSH products #10. MeSH descriptor: [Emergency Medicine] explode all trees #11. MeSH descriptor: [Emergency Medical Technicians] explode all trees #12. MeSH descriptor: [Emergency Nursing] explode all trees #13. #7 or #8 or #9 or #10 or #11 or #12 #14. ("pain management*" or "pain treatment*" or "pain intervention*" or analgesi* or analgaesi* or "analgetic agent*" or "analgesic drug*" or anodynes or antinociceptive or NSAIDs or paracetamol or ketamine or fentanyl or "nitrose oxide" or methoyloxine or morphine or opoid or non-opoid or narcotics or non-narcotics or "regional nerve block*" or "per oral" or peroral or intravanous or "intra venous" or IV or intramuscular or "intra muscular" or "intra nasal" or intranasal or inhalational or "pain reduction" or "pain relief" or oligoanalgesia or "pain intensity" or "pain assessment" or "pain measurement" or effectiveness or "effect management" or "side effect*" or "adverse effect*" or "adverse event*"):ti,ab,kw #15. MeSH descriptor: [Pain Management] explode all trees #16. MeSH descriptor: [Analgesia] explode all trees #17. MeSH descriptor: [Analgesics] explode all trees #18. MeSH descriptor: [Nerve Block] explode all trees #19. MeSH descriptor: [Fentanyl] explode all trees #20. MeSH descriptor: [Ketamine] explode all trees #21. MeSH descriptor: [Morphine] in all MeSH products #22. MeSH descriptor: [Acetaminophen] explode all trees #23. MeSH descriptor: [Drug Administration Routes] explode all trees #24. MeSH descriptor: [Pain Measurement] explode all trees #25. #14 or #15 or #16 or #17 or #18 or #19 or #20 or #21 or #22 or #23 or #24 #26. #6 and #13 and #25 #27. #6 and #13 and #25 with Cochrane Library publication date from Jan 2000 to Dec 2020

**Table 7:** Epistemonikos Search Strategy

(title:(infant* OR newborn* OR child* OR "preschool child*" OR juvenile* OR preschool* OR adolescent* OR pediatric* OR paediatric* OR "young people" OR "young person") OR abstract:(infant* OR newborn* OR child* OR "preschool child*" OR juvenile* OR preschool* OR adolescent* OR pediatric* OR paediatric* OR "young people" OR "young person")) AND (title:(ambulance* OR "transportation of patient*" OR "emergency service*" OR "emergency medical service*" OR EMS OR "emergency health service*" OR "prehospital care" OR prehospital* OR pre-hospital* OR "out of hospital" OR "out of hospital care" OR "emergency medicine" OR "pediatric emergency medicine" OR "paediatric emergency medicine" OR "emergency responder*" OR "first responder*" OR "emergency medical technician*" OR "emergency technician*" OR "emergency practitioner*" OR " emergency medical practitioner*" OR "emergency care practitioner*" OR EMT OR "rescue personnel*" OR "emergency nurse*" OR paramedic*) OR abstract:(ambulance* OR "transportation of patient*" OR "emergency service*" OR "emergency medical service*" OR EMS OR "emergency health service*" OR "prehospital care" OR prehospital* OR pre-hospital* OR "out of hospital" OR "out of hospital care" OR "emergency medicine" OR "pediatric emergency medicine" OR "paediatric emergency medicine" OR "emergency responder*" OR "first responder*" OR "emergency medical technician*" OR "emergency technician*" OR "emergency practitioner*" OR " emergency medical practitioner*" OR "emergency care practitioner*" OR EMT OR "rescue personnel*" OR "emergency nurse*" OR paramedic*)) AND (title:("pain management*" OR "pain treatment*" OR "pain intervention*" OR analgesi* OR analgaesi* OR "analgetic agent*" OR "analgesic drug*" OR anodynes OR antinociceptive OR NSAIDs OR paracetamol OR ketamine OR fentanyl OR "nitrose oxide" OR methoyloxine OR morphine OR opoid OR non-opoid OR narcotics OR non-narcotics OR "regional nerve block*" OR "per oral" OR peroral OR intravanous OR "intra venous" OR IV OR intramuscular OR "intra muscular" OR "intra nasal" OR intranasal OR inhalational OR "pain reduction" OR "pain relief" OR oligoanalgesia OR "pain intensity" OR "pain assessment" OR "pain measurement" OR effectiveness OR "effect management" OR "side effect*" OR "adverse effect*" OR "adverse event*") OR abstract:("pain management*" OR "pain treatment*" OR "pain intervention*" OR analgesi* OR analgaesi* OR "analgetic agent*" OR "analgesic drug*" OR anodynes OR antinociceptive OR NSAIDs OR paracetamol OR ketamine OR fentanyl OR "nitrose oxide" OR methoyloxine OR morphine OR opoid OR non-opoid OR narcotics OR non-narcotics OR "regional nerve block*" OR "per oral" OR peroral OR intravanous OR "intra venous" OR IV OR intramuscular OR "intra muscular" OR "intra nasal" OR intranasal OR inhalational OR "pain reduction" OR "pain relief" OR oligoanalgesia OR "pain intensity" OR "pain assessment" OR "pain measurement" OR effectiveness OR "effect management" OR "side effect*" OR "adverse effect*" OR "adverse event*"))limit 2000–2020	

### Study selection

The results of the conducted search were combined, and duplicate studies were eliminated using the Covidence software. Titles and/or abstracts of studies were screened and carefully read by the four review authors (YA, TS, FH and KS) to identify potentially eligible studies. The full text of these articles was then retrieved and independently reviewed by the same four authors. The articles were included if the two authors agreed on that specific article. Discrepancies were resolved through discussion with a third reviewer (one of the four authors who had not reviewed that specific article).

### Data extraction

We modified a data extraction template from Covidence version 2.0 [27]. One randomly selected article among the included studies was used as a pilot test. Two review authors performed the extraction (YA and TS) and resolved any disagreements through discussion. The general characteristics of the included studies, the name of the administered analgesic drug, the route of administration and the dose were extracted. In addition, the two outcome variables of the review and any age-specific results for small children (< 5 years old) were extracted. An email was forwarded to the respective corresponding authors of the included studies to obtain any additional information and confirm the correctness of the extracted data.

### Assessment of the risk of bias

The Joanna Briggs Institute (JBI) checklist was used to assess the risk of bias for each of the study designs employed in the included articles [28]. The JBI checklists for analytical cross-sectional, prevalence, case series and cohort study designs were used. The level of evidence (LOE) was also classified according to the evidence evaluation worksheet by the International Liaison Committee on Resuscitation for therapeutic interventions (Table 8) [29]. Two authors (YA and KS) independently conducted a risk of bias assessment and a third author (FH) resolved any disagreements. No article was excluded on the basis of these assessments.

**Table 8:** Levels of Evidence (LOE) for Studies of Therapeutic Interventions

LOE 1	Randomised Controlled Trials (or meta-analyses of RCTs)
LOE 2	Studies using concurrent controls without true randomisation (e.g. “pseudo”-randomised)
LOE 3	Studies using retrospective controls
LOE 4	Studies without a control group (e.g. case series)
LOE 5	Studies not directly related to the specific patient/population (e.g. different patient/population, animal models, mechanical models, etc.)

### Data synthesis

Due to the heterogeneity of outcome variables, we performed a textual narrative analysis of the findings from each of the included studies. We structured our synthesis based on the characteristics of the studies and the types of drugs they included.

## Results

**Fig. 1:**
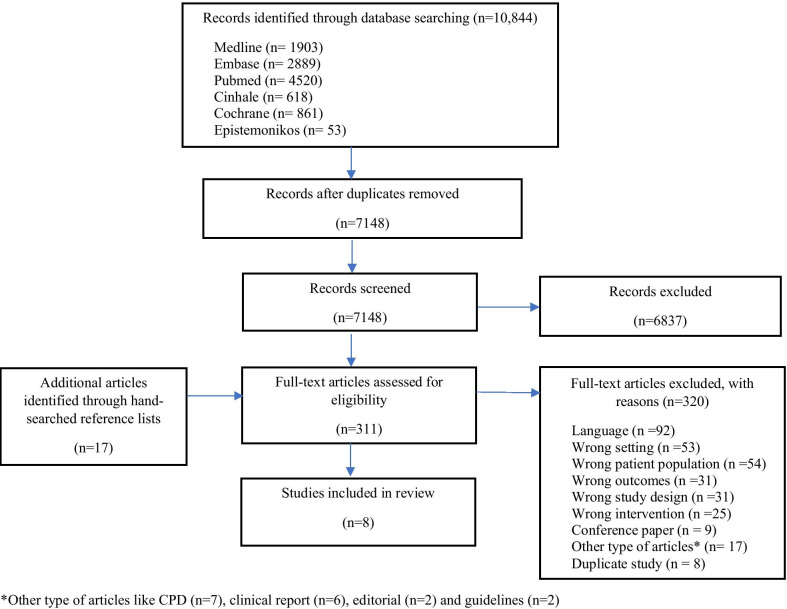
PRISMA flow chart indicating the number of identified and included articles

We imported a total of 10,844 studies from six electronic databases. After removing duplicates (n = 3696), 7,148 titles/abstracts were screened. Of these, we reviewed 311 full-text studies. We also reviewed an additional 17 full-text studies identified from hand-searched reference lists. In all, 328 full-text articles comprising 320 studies were excluded, and eight studies [30–37] that met the eligibility criteria were included in the review. The selection process and grounds for exclusion are presented in a PRISMA flowchart below (Fig. 1).

### Characteristics of the included studies

According to the evidence evaluation worksheet developed by the International Liaison Committee on Resuscitation for therapeutic interventions, the current review found level three and four evidence only, despite using a broad search strategy. Among the eight included studies, six were cross-sectional studies while the remaining two were case series and cohort studies, respectively. All studies were conducted in high-income countries; five in Australia [30, 31, 33, 34, 36] and three in Europe [32, 35, 37]. The publication year ranges from 2006 to 2017. The studies involved a total of 71,674 study participants. Six studies examined the paediatrics age group only. The other two [32, 34] included both adults and paediatrics (both had a separate report for the paediatric age group). The overall characteristics of the included studies are presented in Table 9.

**Table 9:** Characteristics of the included studies

Author, year, country	Funding, conflict of interest	Study design, (LOE)	Age groups, Median age in years (IQR)	Total No. of Patients	The commonly reported etiology	Pain Assessment Tool	Drug name, route, initial dose	Initial pain score (Among the recorded pain scale)	Reduction of pain severity	Adverse event
Bredmose P. 2009 [35], UK	Reported, None	Retrospective database review (LOE 4)	Pediatrics, 10 (0–15)**	164	Trauma (road traffic accident, burns, and fall)	NR*	Ketamine, IV/IM, mean dose 1.0 mg/kg (range 0.1–5.8),	NR*	NR*	Desaturation (< 4%)
Karlsen, A. P. H. 2013 [32], Denmark	Reported, None	Prospective data review (LOE 4)	Adult and pediatrics, 15 (13–16)	Only pediatrics (63)	Nonspecific	Numeric rating scale	Fentanyl, intranasal, 50 µg (mean cumulative dose of 114 µg),	Median initial pain score (IQR) 8 (7–9)	Median reduction in pain score (IQR) 4 (2–5)	No serious adverse events. Commonly observed adverse events were hypotension, nausea, and vomiting, transient GCS reduction, vertigo, fatigue, worsening of abdominal pain, and rash
Murphy, A. P. 2017 [37], Ireland	Reported, None	Prospective chart review (LOE 4)	Pediatrics, 11 (7–13)	94	Trauma	Either FLACC, the Wong-Baker Faces, or the Verbal numerical rating scale	Fentanyl, intranasal, mean (SD) 50 µg (± 10 µg)	Median initial pain score (IQR) 10 (8–10)	Median (IQR) pain score reduction of 4 (*P* < 0.001; [2–6	No observed adverse events
Bendall, J. C 2011 [36], Australia	NR*, None	Retrospective chart review (LOE 4)	Pediatrics, 13 (11–14)	3312	Trauma, abdominal pain, back pain, and other (nonspecific)	Verbal numerical rating scale	Morphine, IV, 0.1 mg/kg for children aged 5-12 years and 2.5–5.0 mg for children > 12yrs, Fentanyl, intranasal, 45-60 mg (1–5 yrs), 60-75 mg (6-12yrs), 180 mg (13-15yrs), Methoxyflurane, inhalational, (0.2%-0.4%) 3 ml	Median initial pain score (IQR) 8(7–10)	Median pain score reduction (IQR) 5(3–6)	NR*
Jennings, P. A. 2015 [33], Australia	NR*, NR*	Retrospective data review (LOE 4)	Pediatrics 11 (9–13)	15,016	Musculoskeletal injury, and others (including medical cause)	Verbal numerical rating scale, the Wong-Baker Faces pain scale, FLACC, and use of adjectives	Methoxyflurane, inhalational, NR* Fentanyl, intranasal/IV, NR* Morphine, IV, NR*	Median initial pain score (IQR) 7 (5–8)	Median pain score reduction (IQR) 4 (2–6)	NR*
Lord, B. 2016 [31], Australia	NR*, None	Retrospective database review (LOE 4)	Pediatrics 10 (5–12	38,167	Musculoskeletal (commonly from fall), burns, poisoning, other trauma, cardiac and other causes	Verbal numerical rating scale, the Wong-Baker Faces pain scale, FLACC, and use of adjectives	Fentanyl, intranasal, NR* Methoxyflurane, inhalational, NR* Morphine, IV (65%), NR*	Median initial pain score (IQR) 3 (2–7)	Median pain score reduction (IQR) 2 (0–5)	NR*
Babl F.E. 2006 [30], Australia	NR*, None	Prospective case series (LOE 4)	Pediatrics 11 (8.6–13.5)	105	Extremity injury, abdominal pain, head/facial injury, burn, and multi-trauma	Verbal numerical rating scale	Methoxyflurane, inhaled, 3 ml (91.4%) and 6 ml (8.6%)	Mean of 7.9 (95% CI 7.5–8.3)	Mean of 4.5 (95% CI 3.9–5.0) at 2-5 min and 3.2 (95% CI 2.8–3.7)	No major adverse events. Minor adverse events like drowsiness, hallucinations, vomiting, confusion/dyscoordination, dizziness, cough, headache, and others. No reported renal impairment
Jacobs Ian G. 2010 [34], Australia,	Reported, None	Retrospective Cohort (LOE 3)	Adult and pediatrics, No specific pediatric median age	Only pediatrics (14,753)	Nonspecific	NR*	Methoxyflurane, inhaled, (0.3%) 3 ml	NR*	NR*	No increased risk of disease

^*^NR (not reported) **Only range (median is not reported)

### Types of drugs

Six studies [30–33, 36, 37] examined the effectiveness of the reported analgesic drugs while two did not address this outcome [34, 35]. Furthermore, five studies [30, 32, 34, 35, 37] assessed the safety of the drugs, while the other three focused solely on their effectiveness [31, 33, 36].

#### Effectiveness and safety of intranasal Fentanyl as a single analgesic drug

Five of the studies [31–33, 36, 37] evaluated fentanyl as a single analgesic drug in children. Jennings describes fentanyl administration via both intranasal and IV routes (the results were not specified separately for each route of administration) [33], while the other studies addressed fentanyl administration via the intranasal route only. Each of the five studies identified fentanyl as an effective analgesic drug. Of these, two studies [32, 37] found that Intranasal Fentanyl (INF) given at a dose of 50 µg as a single dose (mean cumulative dose of 114 µg) [32] and a total of 1.5 µg/kg (initial mean (SD) dose of 50 (± 10)µg) [37] had no serious adverse events. The most common minor adverse events reported due to the administration of fentanyl are presented in Table 9. The dosage and any adverse events of fentanyl were not described in the other three studies [31, 33, 36]

#### Effectiveness and safety of Morphine as a single analgesic drug

Among the eight included studies, three investigated the use of morphine [31, 33, 36], all of which described the effectiveness of morphine administration in pain reduction. None of these studies stated the drug dosages or any adverse events.

In the study by Lord [31], 65% of morphine administration was via the IV route while other routes of administration were not described. The other two studies [33, 36] examined morphine administration via the IV route only. These two studies also compared the effect of morphine and fentanyl on pain reduction, and both found that morphine had an equivalent effect to fentanyl in pain reduction.

#### Effectiveness and safety of inhalational Methoxyflurane as a single analgesic drug

A total of five studies [30, 31, 33, 34, 36] addressed the use of inhalational Methoxyflurane (IHM). Of these, three studies [31, 33, 36] examined the effectiveness of the drug, while the other two [30, 34] focused on adverse events related to IHM.

A case series conducted over a time span of eight months in Australia identified a mean pain reduction from the initial pain score of 7.9–3.2 after 10 min of IHM administration [30]. In this study, about 91.4% of children received a single dose of 3 ml IHM while the others received two doses. In addition, only ten (9.5%) patients received additional IV morphine after the IHM administration had commenced. No serious adverse events had been caused by the IHM administration. About 33.3% and 8% of children under and over five years, respectively, developed deep sedation after receiving IHM. None of these deeply sedated patients had received additional IV morphine. They had immediately regained full consciousness within minutes as methoxyflurane administration was discontinued, and no further measures were needed. In addition, no renal impairment was reported from methoxyflurane administration. The most common minor adverse events found in this study are presented in Table 9.

One cohort study [34] conducted a separate analysis of 594 patients under the age of 12 years who received methoxyflurane, all of whom received a single IHM dose of 3 ml (0.3%). This study did not address the effectiveness of the drug. However, it found no observed increased risk of disease occurrence following methoxyflurane administration when compared to a similar group of patients who did not receive methoxyflurane. The investigated outcome variables among the exposed and control group were the presence of ischemic heart disease, diabetes, renal disease, cancer and hepatic diseases.

The other three retrospective cross-sectional studies conveyed the effectiveness of methoxyflurane in pain reduction [31, 33, 36]. In addition, Bendall found that IHM had less analgesic effect when compared to morphine, fentanyl and combined agents (AOR 0.52; 95% CI 0.36–0.74) [36]. In contrast, another study [33] reported that methoxyflurane had the greatest odds of achieving clinically meaningful pain reduction when compared to morphine and fentanyl (AOR 5.3; 95% CI 4.8–5.9). The drug doses and/or any adverse events were not described in any of the three retrospective cross-sectional studies.

#### Effectiveness and safety of Ketamine as a single analgesic drug

Only one retrospective database review examined the use of ketamine in children below the age of 16 [35]. The mean administered drug dose was 1.0 mg/kg (ranges from 0.1 to 5.8 mg/kg). The route of administration was IV (86%) and intramuscular (IM) (14%). A majority (68%) of patients also received a mean dose of 0.1 mg/kg midazolam as a co-drug. The study did not look at the effectiveness of the drug. No deaths or any implementation of basic airway manoeuvres had occurred due to ketamine administration. In all, only one adverse event had been recorded, which was desaturation (desaturation < 4%) in only four (2.4%) patients. Furthermore, the study did not find desaturation in children younger than three years of age after receiving an analgesic dose of ketamine.

#### Effectiveness and safety of combination analgesic drugs

Three studies examined the effectiveness of combination analgesic drugs [31, 36, 37]. All three identified the effectiveness of the use of combination drugs. None of these studies examined adverse events from the use of combination drugs.

The first study reported that intranasal fentanyl in combination with paracetamol ± ibuprofen ± inhaled nitrous oxide had a 79% effectiveness in pain reduction [37]. The second study showed that a combination of the three drugs (morphine, fentanyl and methoxyflurane) had a median pain score change of 4 (IQR 3–6) [31]. The third study identified that the use of a combination of more than one drug from the three analgesics (morphine, fentanyl and methoxyflurane) had a statistically significant higher median pain score difference (median pain score difference of 6 (IQR 4–7)) compared to use of the drugs independently. However, there was no statistical evidence suggesting that combination drugs were more effective than morphine or fentanyl alone after controlling for factors such as age and gender [36].

### Risk of bias

The JBI critical appraisal checklist was used to assess the risk of bias. The JBI checklists for analytical cross-sectional, prevalence, case series and cohort study designs were used. Nine questions were assessed using the prevalence study design checklist, eight in the analytical, ten in the case series and eleven in the cohort study design. The findings of the assessment are provided in Table 10.

**Table 10:** Risk of bias assessment findings based on the respective research designs checklist of the JBI tool

Prevalence Cross-Sectional:												
Study ID	1. Was the sample frame appropriate to address the target population?	2. Were study participants sampled in an appropriate way?	3. Was the sample size adequate?	4. Were the study subjects and the setting described in detail?	5. Was the data analysis conducted with sufficient coverage of the identified sample?	6. Were valid methods used for the identification of the condition?	7. Was the condition measured in a standard, reliable way for all participants?	8. Was there appropriate statistical analysis?	9. Was the response rate adequate, and if not, was the low response rate managed appropriately?	Overall appraisal		
Bredmose P. [35] 2009	Yes	Yes	Yes	No	Yes	Yes	Unclear	Yes	Not applicable	Include		
Karlsen, A. P. H. [32] 2013	Yes	Unclear	Unclear	Yes	Yes	Yes	Yes	Yes	Yes	Include		
Murphy, A. P. [37] 2017	Yes	Yes	Unclear	Yes	Yes	Yes	Unclear	Yes	Yes	Include		

Analytical Cross-Sectional:												
Study ID	1. Were the criteria for inclusion in the sample clearly defined?	2. Were the study subjects and the setting described in detail?	3. Was the exposure measured in a valid and reliable way?	4. Were objective, standard criteria used for measurement of the condition?	5. Were confounding factors identified?	6. Were strategies to deal with confounding factors stated?	7. Were the outcomes measured in a valid and reliable way?	8. Was appropriate statistical analysis used?	Overall appraisal			
Bendall, J. C [36] 2011	Yes	Yes	Unclear	Yes	Yes	Yes	Yes	Yes	Include			
Jennings, P. A [33] 2015	Unclear	Yes	Unclear	Unclear	No	Unclear	Yes	Yes	Include			
Lord, B. [31] 2016	Yes	Yes	Unclear	Yes	No	No	Yes	No	Include			

**Case Series:**												
**Study ID**	**1. Were there clear criteria for inclusion in the case series?**	**2. Was the condition measured in a standard, reliable way for all participants included in the case series?**	**3. Were valid methods used for identification of the condition for all participants included in the case series?**	**4. Did the case series have consecutive inclusion of participants?**	**5. Did the case series have complete inclusion of participants?**	**6. Was there clear reporting of the demographics of the participants in the study?**	**7. Was there clear reporting of clinical information of the participants?**	**8. Were the outcomes or follow-up results of cases clearly reported?**	**9. Was there clear reporting of the presenting site(s)/clinic(s) demographic information?**	**10. Was statistical analysis appropriate?**	**Overall appraisal**	
Babl F.E. [30] 2006	Yes	Unclear	Yes	Yes	Yes	Yes	Yes	Yes	Yes	Yes	Include	

**Cohort Design:**												
**Study ID**	**1. Were the two groups similar and recruited from the same population?**	**2. Were the exposures measured similarly to assign people to both exposed and unexposed groups?**	**3. Was the exposure measured in a valid and reliable way?**	**4. Were confounding factors identified?**	**5. Were strategies to deal with confounding factors stated?**	**6. Were the groups/participants free of the outcome at the start of the study (or at the moment of exposure)?**	**7. Were the outcomes measured in a valid and reliable way?**	**8. Was the follow-up time reported and sufficient to be long enough for outcomes to occur?**	**9. Was follow up complete, and if not, were the reasons to loss to follow up described and explored?**	**10. Were strategies to address incomplete follow up utilized?**	**11. Was appropriate statistical analysis used?**	**Overall appraisal**
Jacobs Ian G. [34] 2010	Yes	Yes	Yes	Unclear	Unclear	Unclear	Yes	Yes	Not applicable	Unclear	Yes	Include

## Discussion

The present systematic review on the effectiveness and safety of analgesic drugs used in prehospital pain relief in children identified a total of eight articles. Most of these articles concerned analgesic drugs such as fentanyl (intranasal/IV), morphine (IV), methoxyflurane (inhalational) and ketamine (IV/IM). The studies examine the effects of fentanyl (intranasal/IV), morphine (IV) and methoxyflurane (inhalational), and all of the analgesic drugs were found to be effective. Adverse events of intranasal fentanyl, inhalational methoxyflurane and IV/IM ketamine were also described. However, none of these drugs were found to have serious adverse events.

The Italian Intersociety Recommendations on pain management in emergency settings stated that ‘the ideal prehospital analgesic should be easy to use, safe, effective, and have a predictable dose–response relationship with rapid onset and a short duration of action’ [38]. Our results show that both intranasal fentanyl and inhalational methoxyflurane are effective and safe to administer both as single drugs and/or in combination with other examined analgesic drugs. Similarly, several studies conducted in different acute care settings support that intranasal fentanyl and inhalational methoxyflurane are effective analgesic drugs with no serious adverse events [19, 39–43]. It has been suggested that these drugs are easy to administer and have a rapid onset and short duration of action [42, 44, 45]. Moreover, the present review found that fentanyl has an equivalent effect to morphine. A randomised control trial conducted in Australian emergency departments also found similar results [39]. Therefore, intranasal fentanyl and inhalational methoxyflurane seem to be the drugs of choice due to their ease of administration, rapid onset, short duration of action, effect and safety profile for children's pain relief in prehospital settings. However, it can be difficult to administer inhalational methoxyflurane in non-cooperative children and in cases involving facial trauma. Similarly, contraindications for nasal drug administrations could be a limitation to administering intranasal fentanyl.

Morphine (IV) was identified in the present systematic analysis as an effective analgesic drug, but the safety of the drug was not evaluated in any of the studies included in this review. This is supported by previous results, and IV morphine has traditionally been reported as the gold standard drug for acute pain relief in acute care settings [40, 46, 47]. Furthermore, the findings support that analgesic doses of ketamine (IV/IM) are safe to use in acute care settings [48–50]. Although we could not find any studies that address the effectiveness of ketamine for the purpose of this review, previous studies have identified ketamine as an effective analgesic drug in acute care settings [48–50]. However, difficulties concerning IV access, anxiety related to painful IM injection and prolonged prehospital time are obvious limitations in IV/IM morphine and ketamine administration in the prehospital pain management of children [13, 40, 41].

The current review did not identify any serious adverse events from the analgesic drugs included in the studies. However, previous studies have demonstrated that opioids (morphine and fentanyl) have adverse events such as respiratory depression, apnoea, sedation, bradycardia and gastrointestinal dysmotility [51, 52]. There is also a fear of sedation, respiratory depression, renal and hepatic failure related to methoxyflurane use [53, 54]. In addition, Ketamine has dose-dependent adverse events such as sedation, hypoxia, laryngospasm, hypersalivation, nausea and vomiting [55, 56]. Hence, to minimise the potential risk due to these adverse events, opioids, methoxyflurane and ketamine should always be administered with caution. Naloxone for an opioid antagonist and/or airway opening and ventilatory devices should always be ready on hand [57].

### Limitations of the included studies and the current review

Most of the studies included in this review were based on retrospective chart reviews. Poor data recording, underreport biases and selection biases are the major challenges of such designs. The training level, scope, and expertise of the care providers could also vary accordingly. It is unclear whether such variations would affect the effectiveness and safety of the included drugs. It would be good to study this in the future research.

Our systematic review has some limitations. Firstly, the systematic literature searches were limited by publication year (the past 20 years from 2000 to 2020) and language (English, Danish, Norwegian or Swedish). This was due to the purpose of having current pharmacological prehospital pain management modalities and due to the authors’ language capabilities and expenses. Secondly, the corresponding authors of four of the included studies [32, 34, 36, 37] did not respond to requests for further information when contacted.

Except in combination with opioids, the authors were not able to find studies that examine the effectiveness and safety of any other common analgesic drugs such as acetaminophen and Non-Steroidal Anti-Inflammatory Drugs (NSAIDs), which could be given for mild to moderate pain [43, 51, 52]. We were also unable to find studies related to the effects and safety of prehospital use of nitrous oxide (Entonox) and nerve block drugs in children.

## Conclusions

Our systematic review revealed that fentanyl (intranasal/IV), morphine (IV), methoxyflurane (inhalational) and combination drugs are effective analgesic drugs for children in prehospital settings. No serious adverse events were reported in the administration of intranasal fentanyl, inhalational methoxyflurane or IV/IM ketamine. Intranasal fentanyl and inhalational methoxyflurane seem to be the preferred drugs for children in pre-hospital settings due to their ease of administration, similar effect and safety profile when compared to other analgesic drugs. However, caution must be shown in reaching this conclusion since the included studies’ level of evidence (LOE) was level three and four only.

This systematic review found that there is a paucity of high-level evidence on children’s prehospital pain management. Furthermore, all of the studies included were conducted solely from the perspectives of high-income countries. Well-designed comprehensive studies that include the context of low- and middle-income countries should also be conducted. In addition to single analgesic drugs, multimodal analgesia also needs further analysis in future studies of prehospital pain management in children.

## Data Availability

All data generated or analysed during this study are included in this published article.

## Abbreviations

CINAHL: Cumulative Index of Nursing and Allied Health Literature
CPDs: Continuing Professional Developments
DIKU: Norwegian Agency for International Cooperation and Quality Enhancement in Higher Education
ED: Emergency Department
Embase: Excerpta Medica Database
FDA: Food and Drug Administration
IHM: Inhalational Methoxyflurane
IM: Intramuscular
INF: Intranasal Fentanyl
IV: Intravenous
JBI: Joanna Briggs Institute
LOE: Level of Evidence
Medline: Medical Literature Analysis and Retrieval System Online
MeSH: Medical Subject Headings
NORPART: Norwegian Partnership Programme for Global Academic Cooperation
NSAIDs: Non-steroidal Anti-inflammatory Drugs
PRISMA: Preferred Reporting Items for Systematic Reviews and Meta-analyses
PROSPERO: International Prospective Register of Systematic Review
